# Association Between Changes in Plasma Capecitabine Concentrations and Adverse Events in the Treatment of Colorectal Cancer

**DOI:** 10.7759/cureus.71341

**Published:** 2024-10-12

**Authors:** Yasuhiro Hashimoto, Yoichiro Yoshida, Teppei Yamada, Gumpei Yoshimatsu, Fumihiro Yoshimura, Suguru Hasegawa

**Affiliations:** 1 Gastroenterological Surgery, Fukuoka University Hospital, Fukuoka, JPN

**Keywords:** 5-fluorouracil, adverse event, capecitabine, chemotherapy, colorectal cancer

## Abstract

Background

Therapeutic drug monitoring (TDM) is an effective approach to improving the efficacy of drugs with a narrow therapeutic index and high toxicity. TDM-guided dosing of 5-fluorouracil (5-FU) has been shown to result in superior efficacy and fewer adverse events compared to body surface area (BSA)-based dosing. Therefore, accurate measurement of plasma 5-FU concentrations after capecitabine administration is necessary. Capecitabine is a prodrug of 5-FU and is metabolized to 5-FU in multiple steps in the gastrointestinal tract, liver, and within tumors. To solve the problem of frequent blood draws for TDM, we reduced the number of blood draws to two and examined whether changes in 5-FU concentration correlated with adverse events.

Methods

This study investigated the relationship between the changes in plasma 5-FU concentrations after one and two hours of capecitabine administration in 36 patients and adverse events based on drug concentrations determined after adding 5-NU to the plasma samples. Concentration gradients and adverse events were estimated using the Mann-Whitney test.

Results

The median one- and two-hour plasma 5-FU concentrations were 67.5 (range 5-307) and 85.5 (range 19-246) ng/mL, respectively. The plasma 5-FU concentration gradient, defined as the difference between the one- and two-hour concentrations, was significantly higher in patients with diarrhea and nausea (p = 0.0234 and p = 0.0409, respectively).

Conclusion

The high plasma 5-FU concentration gradient suggests rapid degradation of 5-FU into its metabolites, which may lead to predict intestinal mucosal damage, diarrhea, and nausea.

## Introduction

Ideally, chemotherapeutics should exhibit high therapeutic efficacy while minimizing toxicity. 5-fluorouracil (5-FU) is widely employed as a primary chemotherapeutic agent in various carcinomas, including gastrointestinal cancer [1,2]. Generally, 5-FU doses are adjusted according to the body surface area (BSA) of the patient; however, BSA has been reported to be an inadequate predictor of systemic drug exposure [3,4]. Recently, therapeutic drug monitoring (TDM)-based 5-FU dose titration has been shown to yield better response rates and fewer adverse events compared to the BSA-based 5-FU dose titration [5,6]. TDM is an essential method for optimizing the therapeutic efficacy of drugs with a narrow therapeutic index and high toxicity. However, it is difficult to perform clinically since it requires a large number of blood samples. Clinically, it is desirable to develop a method that requires fewer blood draws. To solve the problem of frequent blood draws for TDM, we reduced the number of blood draws to two and examined whether changes in 5-FU concentration correlated with adverse events.

Prodrugification is another strategy for reducing toxicity and optimizing therapeutic efficacy [7]. Prodrugs can also enhance the parameters of pharmacokinetics and pharmacodynamics. Capecitabine is a precursor of 5-FU and is metabolized to 5-FU through a three-step activation process, reaching maximum plasma concentrations in approximately 1.5 hours [2,8,9]. In the liver, capecitabine is primarily converted to 5'-deoxy-5-fluorocytidine (5'-DFCR) by carboxylesterase, which is not highly active in the intestinal epithelium. Next, 5'-DFCR is sequentially transformed to 5'-deoxy-5-fluorouridine (5'-DFUR) by cytidine deaminase, which is highly expressed in the liver and tumor tissue. Finally, 5'-DFUR is metabolized to 5-FU by thymidine phosphorylase (TP), which is highly expressed in tumor tissue. These consequent metabolic processes reduce the toxicity while increasing the tumor selectivity of 5-FU. Although TDM-based dose adjustment is also expected to improve response rates and reduce adverse events of 5-FU prodrugs [4], there are no large-scale studies on 5-FU dose titration with TDM. For postoperative adjuvant therapy and treatment of advanced recurrence of colorectal cancer, CapeOX, a combination of capecitabine and oxaliplatin, has been developed and has achieved excellent treatment results [10]. The addition of VEGR inhibitors such as bevacizumab for the treatment of advanced recurrence has also shown excellent results [10].

In recent years, various studies have gradually clarified the pharmacokinetics of capecitabine [11,12]. Gieschke et al. (2003) have reported that the plasma levels of 5-FU, 5'-DFUR, and alpha-fluoro-beta-alanine do not necessarily reflect the precise active drug concentrations in healthy and tumor tissues after capecitabine administration [13]. Capecitabine is selectively metabolized to 5-FU via a three-enzyme cascade [14]. During the measurement of plasma 5-FU concentrations after capecitabine administration, inhibitors of these three enzymes have not been used because it has been deemed unlikely that capecitabine would be metabolized to 5-FU in the peripheral blood. Therefore, no clinical study to date has measured plasma 5-FU concentrations in the presence of enzyme inhibitors after administration of capecitabine. 5-nitrouracil (5-NU), which is comparable to 5-FU in structure, suppresses TP and blocks the conversion of 5'-DFUR to 5-FU [15,16]. Capecitabine is thought to be metabolized primarily to 5-FU in tumors [17]. Therefore, 5-FU concentration in plasma is typically evaluated after the oral administration of capecitabine in the absence of metabolic inhibitors, such as 5-NU. However, it was reported that 5'-DFUR continued to be transformed to 5-FU even after the samples were stored at 4°C [18]. Yoshida (2020) et al. validated plasma 5-FU concentrations by adding 5-NU to the plasma samples [18]. The results showed that the plasma 5-FU concentrations changed with time, temperature, and the presence or absence of 5-NU and that they nearly doubled depending on the measurement conditions. It has been found that 5-FU concentration tends to increase under the following conditions at the time of measurement: high room temperature, long time between blood collection and measurement, and the absence of 5-NU. 5-FU blood concentrations measured at room temperature and in the absence of 5-NU are clinically useless because they are higher than actual concentrations. Therefore, accurate determination of plasma 5-FU concentrations is a very important issue. This is the first study in the PubMed search to verify the relationship between changes in plasma 5-FU concentrations and adverse events by determining plasma 5-FU concentrations after the addition of 5-NU to the samples.

## Materials and methods

Patients and eligibility criteria

Between September 2019 and July 2021, 36 patients undergoing capecitabine therapy (capecitabine plus oxaliplatin: 14, capecitabine plus oxaliplatin plus VEGF inhibitors: 22) for colorectal cancer participated in this cohort study, which was conducted in accordance with the ethical guidelines for clinical studies. The institutional review board of Fukuoka University approved the study protocol (approval no. 16.10.02). Informed consent was obtained from all patients. All study procedures were carried out in accordance with the Declaration of Helsinki.

Eligible patients were ≥20 years of age with histologically diagnosed colorectal cancer. The other inclusion criteria were as follows: life expectancy, ≥3 months; Eastern Cooperative Oncology Group performance status, 0-1; neutrophil count, ≥1000/millimeter (mm)^3^; hemoglobin, ≥8.0 gram (g)/decilitre (dL); platelet count, ≥75,000/mm^3^; serum creatinine, ≤1.5 times the upper limit of normal; total bilirubin, ≤2.0 milligram (mg)/dL; aspartate transaminase, ≤100 IU/L (≤200IU/L for patients with metastatic liver tumor); and alanine transaminase, ≤100 International Unit (IU)/liter (L) (≤200IU/L for patients with metastatic liver tumor). Patients fulfilling the following criteria were excluded: serious drug allergy; severe peripheral neuropathy; active infection; uncontrollable hypertension; mechanical or paralytic bowel obstruction; uncontrolled diabetes mellitus; cirrhosis; unstable ischemic heart disease; multiple malignancies within the last five years; ascites, pleural effusion, or pericardial effusion; uncontrolled diarrhea.

Measurement of plasma 5-FU concentrations

In this study, we examined changes in plasma concentrations of 5-FU in capecitabine-treated patients using previously published methods [18]. Plasma 5-FU concentrations peak at one to two hours after oral administration of capecitabine. We indirectly evaluated the metabolic rate by calculating the concentration gradient from plasma 5-FU concentrations at one and two hours after oral administration and examined the association with adverse events. Blood samples (5 milliliters) were collected in tubes containing ethylenediaminetetraacetic acid after one and two hours following the first administration of capecitabine. To reduce the influence of other drugs, plasma concentrations of 5-FU were measured before any other drugs scheduled to be administered on the same day. The samples were collected from all patients at a fixed time following dinner on the first day of hospitalization to minimize the influence of gastrointestinal motility and food. Next, 100 microliters (μL) of 5-NU (15 mM) were added to the samples. After centrifuging the samples at 4°C, the plasma was collected and stored at −80°C. The 5-FU concentration in the plasma was measured immediately after thawing with a homogeneous and competitive nanoparticle immunoassay (My5-FU; Saladax Biomedical, Bethlehem, PA, USA) and a biochemical analyzer (Abbott Architect c4000, Abbott Park, IL, USA), as previously described [18].

Statistical analysis

The statistical analyses were conducted using EZR, a modified version of R (ver 1.4) commander tailored to incorporate commonly used statistical functions in biostatistics. Descriptive statistics were presented as medians (interquartile range), depending on the distribution's normality. Between-group differences were assessed using the Mann-Whitney test for continuous variables and the chi-square test for categorical variables. Fisher's exact test was used for categorical variables. Bonferroni correction was also performed to compare three or more groups. Statistical significance was determined by a probability value (p) of less than 0.05. A heatmap of the correlation coefficients between plasma 5-FU concentration and clinicopathologic factors was generated using Python 3.12. Pandas dataframe.corr() is used to find the pairwise correlation of all columns in the Pandas Dataframe in Python.

## Results

Clinical characteristics of the patients

A total of 36 individuals (21 males and 15 females), with a median age of 67 (61-73 years), participated in this case-control study. The patient demographics are shown in Table 1. The median BSA was 1.57 (1.46-1.69) meter (m)^2^, and the median creatinine clearance (Ccr) was 99.0 (86.3-128.3) ml/min. The primary lesions were colon and rectal cancer in 16 (44%) and 20 (56%) patients, respectively. Chemotherapy included postoperative adjuvant therapy in 12 (33.3%) patients, neoadjuvant therapy in eight (22.2%) patients, and systemic chemotherapy for metastatic cancer in 16 (44.4%) patients. The median dose of capecitabine was 3,000 mg/day. One patient was administered a reduced initial dose of capecitabine due to decreased renal function. All other patients received reduced doses of capecitabine after the onset of adverse events. All patients were treated with oxaliplatin and none with irinotecan. All patients were given the same antiemetics at the same time as chemotherapy administration. The observation period was six months.

**Table 1:** Clinical characteristics of the patients n (%): Sex, primary cancer site, clinical stage, purpose of chemotherapy Median [interquartile range]: Age, BMI, Ccr, Capecitabine dosage n: number of patients; %: percent; kg/m2: kilogram/square meter; ml/min: milliliter/minute; BMI: body mass index; BSA: body surface area; Ccr: creatinine clearance

Clinical characteristics		n = 36
Sex	Male	21 (58.3%)
	Female	15 (41.7%)
Age (years)		67.0 [61.0-73.0]
BMI (kg/m²)		21.2 [14.7-23.5]
BSA (m²)		1.57 [1.46-1.69]
Ccr (ml/min)		99.0 [86.3-128.3]
Primary cancer site	Colon (Right side: 8, Left side: 8)	16 (44%)
	Rectum	20 (56%)
Clinical Stage	Ⅰ	1 (2.8%)
	Ⅱ	5 (13.9%)
	Ⅲ	20 (55.6%)
	Ⅳ	10 (27.8%)
Purpose of chemotherapy	Adjuvant	12 (33.3%)
	Neoadjuvant	8 (22.2%)
	Metastatic	16 (44.4%)
Capecitabine dosage (mg/day)		3,000 [3,000-3,000]

Blood concentrations and concentration gradients of 5-FU at one and two hours

**Figure 1:**
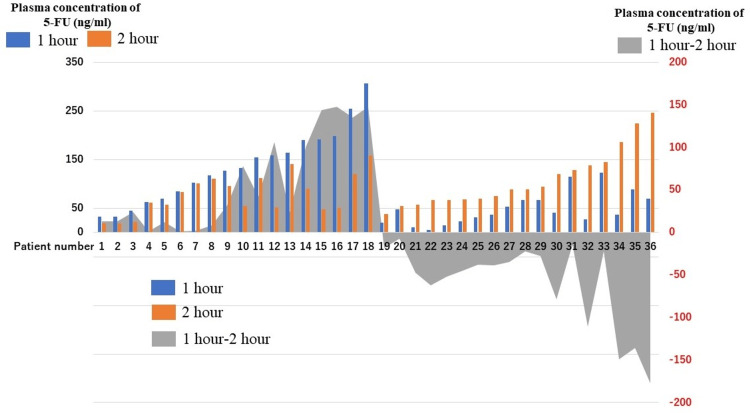
Plasma concentrations and concentration gradient of 5-FU at 1 and 2 hours after capecitabine administration Figure 1 was created to visualize individual differences in the plasma 5-FU concentrations. The plasma 5-FU concentrations at one (blue) and two (orange) hours after the oral administration of capecitabine for each patient. The difference between the one-hour value and the two-hour value is graphed as a concentration gradient (Gray). 5-FU: 5-fluorouracil.

The plasma 5-FU concentrations were measured after the addition of 5-NU to the samples. Figure 1 shows the plasma 5-FU concentrations at one and two hours after the oral administration of capecitabine. The mean plasma 5-FU concentrations were 67.5 (range, 5-307) and 85.5 (range, 19-246 ng/mL) at one and two hours, respectively. The subtraction of the one-hour value from the two-hour value is graphed as a concentration gradient. No significant differences in the plasma 5-FU concentration were found between patients with and without liver metastases. Table 2 shows the rates and grades of adverse events in the overall cohort. The following adverse events were evaluated: diarrhea, stomatitis, nausea, anorexia, fatigue, hand-foot syndrome, constipation, leukopenia, neutropenia, anemia, and thrombocytopenia.

**Table 2:** Adverse events during chemotherapy CTC-AE: Common terminology criteria for adverse events %: percent

Adverse events (CTC-AE)	All grade	Grade1	Grade2	Grade 3
Diarrhea	11(30.6%)	8 (22.2%)	2 (5.6%)	1(2.8%)
Stomatitis	8 (22.2%)	8 (22.2%)	０	0
Nausea	14 (38.9%)	13 (36.1%)	1(2.8%)	0
Loss of appetite	14 (38.9%)	9 (25.0%)	5 (13.8%)	0
Fatigue	18 (50.0%)	14 (38.9%)	4 (11.1%)	0
Hand–foot syndrome	14 (38.9%)	10 (27.8%)	4 (11.1%)	0
Constipation	6 (16.6%)	3 (8.3％)	3 (8.3％)	0
Leukopenia	14 (38.9%)	10 (27.8%)	4 (11.1%)	0
Neutropenia	14 (38.9%)	7 (19.4%)	5 (13.8%)	2 (5.6%)
Anemia	14 (38.9%)	10 (27.8%)	2 (5.6%)	2 (5.6%)
Thrombocytopenia	12 (33.3%)	8 (22.2%)	2 (5.6%)	2 (5.6%)

Relationship between 5FU blood concentration and adverse events

Table 3 shows the relationship of the observed adverse events with plasma 5-FU concentrations. Plasma concentrations were evaluated using the higher of the measured 1H and 2H values. There was no significant difference in any of the adverse events according to plasma 5-FU concentration.

**Table 3:** Relationship between plasma 5-FU concentration and adverse events Concentrations are expressed as median (interquartile range). The differences between groups were estimated using the Mann-Whitney test for continuous variables.

Adverse events		5-FU blood concentration (ng/ml)	p-value
Diarrhea	(－)	94 [64.5–149.5]	
	(＋)	129 [69–192]	0.363
Stomatitis	(－)	95.5 [32–307]	
	(＋)	137 [38–187.5]	0.361
Nausea	(－)	91.0 [67–149.7]	
	(＋)	131 [60.5–187]	0.604
Loss of appetite	(－)	123.5 [60.7–159.5]	
	(＋)	95.5 [68.5–187.5]	0.884
Fatigue	(－)	132.5[60.7–193.5]	
	(＋)	95.5 [60.7–136]	0.384
Hand–foot syndrome	(－)	119.0 [68.7–139.7]	
	(＋)	142.5[64.5–229.5]	0.299
Constipation	(－)	110.5 [67.4–169.5]	
	(＋)	104 [64.5–172.5]	0.99
Leukopenia	(－)	111.5 [66.5–148.2]	
	(＋)	106 [67–20.5.]	0.446
Neutropenia	(－)	95.5 [65.7–143]	
	(＋)	131.5 [73–205.5]	0.194
Anemia	(－)	127.0 [69–186]	
	(＋)	94 [41.5–148.5]	0.157
Thrombocytopenia	(－)	95.5 [63.3–151.7]	
	(＋)	126.5 [70.5–190.5]	0.392

The patients were categorized into those with high plasma 5-FU concentrations at one hour (n = 18) and those with high plasma 5-FU concentrations at two hours (n = 18), to investigate the relationship between the adverse events that appeared. Table 4 shows the detailed patient background characteristics of the two groups. There were no significant differences in patient characteristics such as age, sex, BSA, Ccr, and capecitabine dosage between the two groups. Furthermore, the rates of adverse events were not significantly different between the two groups (data not shown).

**Table 4:** Clinical characteristics of patients categorized according to the timepoint with the higher plasma 5-FU concentration after capecitabine administration The differences between groups were estimated using the Mann-Whitney test for continuous variables and the chi-square test for categorical variables. 1H: 1 hour, 2H: 2 hours 1H > 2H: 5-FU concentration one hour after capecitabine administration is higher than two hours after administration. 2H > 1H: 5-FU concentration two hours after capecitabine administration is higher than that one hour after administration. BMI: Body mass index, BSA: Body surface area, Ccr: Creatinine clearance

Clinical characteristics		Time after capecitabine administration when 5FU blood levels were high		p-value
		１H > 2H (n＝18)	2H > 1H (n＝18）	
Sex	Male	11 (61.1%)	10 (55.6%)	0.99
	Female	7 (38.9%)	8 (44.4%)	
Age		65 (53–73)	68 (62–74)	0.486
BMI		21.6 (19.2–23.4)	20.9 (19.7–25.1)	0.924
BSA (m²)		1.58 (1.44–1.73)	1.56 (1.46–1.70)	0.788
Ccr (ml/min)		98.6 (83.6–128.2)	99.0 (83.2–151.5)	0.849
Primary cancer site	Colon	6 (33.3%)	10 (55.6%)	0.315
	Rectum	12 (66.7%)	8 (44.4%)	
Clinical stage	Ⅰ	0 (0.0%)	1 (5.6%)	0.719
	Ⅱ	3 (16.7%)	2 (11.1%)	
	Ⅲ	9 (50.0%)	11 (61.1%)	
	Ⅳ	6 (33.3%)	4 (22.2%)	
Purpose of chemotherapy	adjuvant	6 (33.3%)	6 (33.3%)	0.768
	neoadjuvant	5 (27.8%)	3 (16.7%)	
	metastatic	7 (38.9%)	9 (50.0%)	
Capecitabine dosage (mg/day)		3000 (3000–3150)	3000 (2400–3000)	0.311

Concentration gradient between 1H and 2H values of 5-FU blood concentration and adverse events

Table 5 shows the relationship of adverse events with the plasma 5-FU concentration gradient. The plasma 5-FU concentration gradient was significantly greater in patients with diarrhea than in those without diarrhea (23.0 [−14 ~ 144] vs. −22.0 [−54.5 ~ −13], p = 0.0234) and those with nausea than in those without nausea (13.0 [−19~−101.5] vs. −25.0 [−54.5 ~ 1.8], p = 0.0409).

**Table 5:** Relationship between plasma 5-FU concentration gradient between one and two hours and adverse events Concentrations are expressed as median (interquartile range). The differences between groups were estimated using the Mann-Whitney test for continuous variables. 1H: 1 hour, 2H: 2 hours, (-): negative, (+): positive, ~: range

Adverse events		Concentration gradient between 1H and 2H values of 5-FU blood concentration (ng/ml)	p-value
Diarrhea	(－)	−22.0[−54.5~ −13]	
	(＋)	23.0[−14~ 144]	0.0234
Stomatitis	(－)	5.0[−43.5~ 39.5]	
	(＋)	−25.0[−121.3~ −15]	0.138
Nausea	(－)	−25.0[−54.5~ 14.8]	
	(＋)	13.0[−19~ 101.5]	0.0409
anorexia	(－)	1.0[−32.3~ 34.5]	
	(＋)	−28.5[−54.5~ 29.3]	0.363
Fatigue	(－)	6.5[−63~ 49]	
	(＋)	−18.0[−40.5~ 20.3]	0.646
Hand-foot syndrome	(－)	−3.0[−35.8~ 23.6]	
	(＋)	−5.0[−80.5~ 101.5]	0.758
Constipation	(－)	1.0[‐39.7~ 26]	
	(＋)	−30.5[−50.8~ 92.6]	0.702
Leukopenia	(－)	−6.5[−39.8~ 23.6]	
	(＋)	−3.0[‐48.6－ 108.8]	0.808
Neutropenia	(－)	−6.5[−49.3~ 23.3]	
	(＋)	−3.0[‐41~ 108.8]	0.604
Anemia	(－)	−7.0[−39~ 42]	
	(＋)	8.0[−46~ 23.5]	0.974
Thrombocytopenia	(－)	1.0[−31.8~ 23.8]	
	(＋)	−33.0[−70.5~ 99]	0.675

Heat map of correlation coefficients between 5-FU blood levels and clinicopathological factors

**Figure 2:**
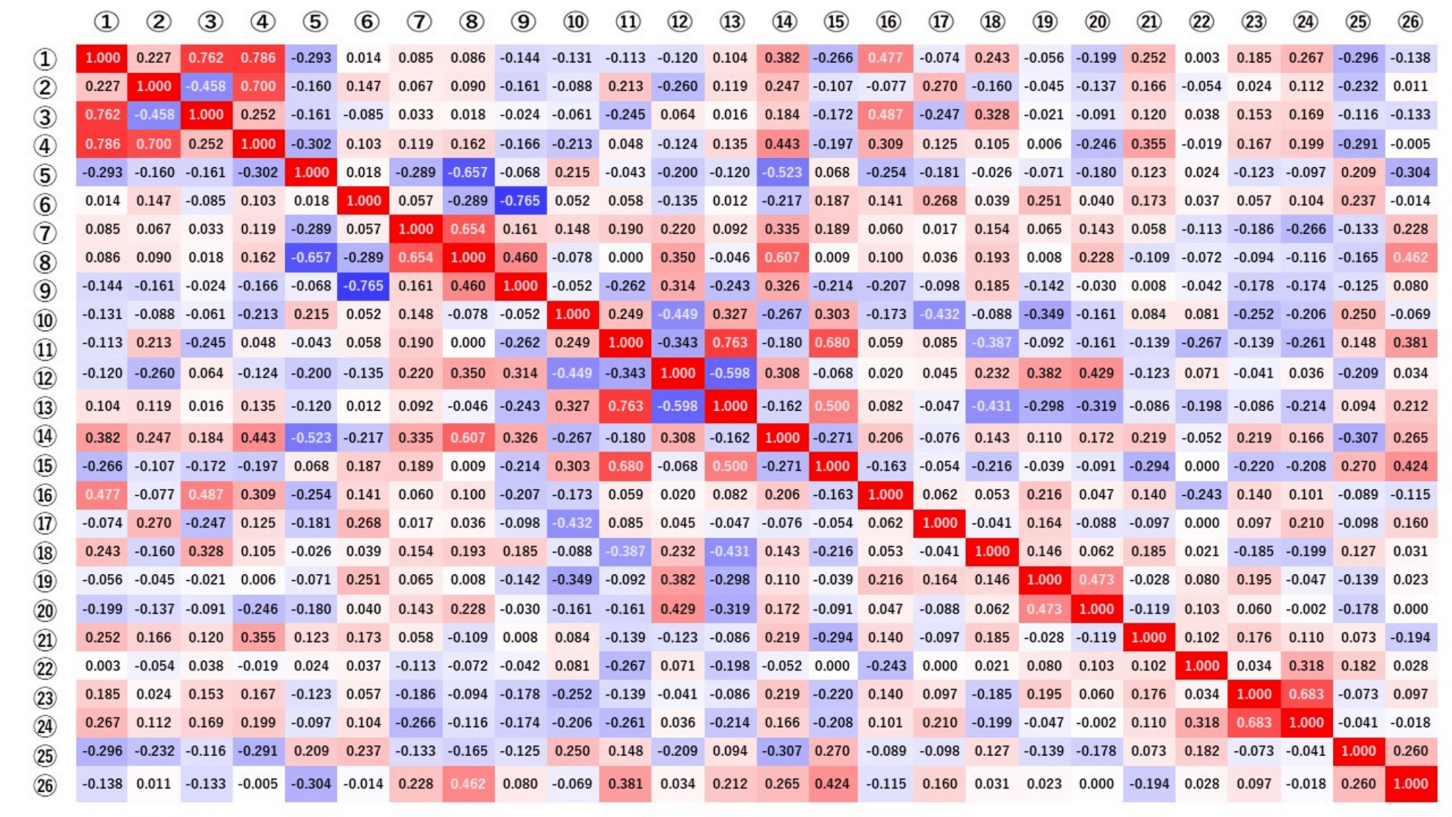
Heatmap of the correlation coefficients between plasma 5-FU concentration and clinicopathologic factors The heat map is created so that a correlation coefficient of 1 is red and -1 is blue. The numbers in the figure represent correlation coefficients. The heatmap of correlation coefficients among the clinicopathologic factors also demonstrated that the plasma 5-FU concentration gradient correlated with diarrhea and nausea. Adverse events correlated with plasma 5-FU concentration were plasma 5-FU concentration after one hour and diarrhea, and concentration gradient and diarrhea/nausea. ①:plasma 5-FU Concentration after one hour; ②: plasma 5-FU Concentration after two hours; ③: ①ｰ②Concentration gradient; ④: Plasma 5-FU concentration at the highest of 1 or 2 hours;　⑤: Sex; ⑥: Age; ⑦: Body mass index; ⑧: Body surface area; ⑨: Creatinine clearance; ⑩: clinical Stage; ⑪: Purpose of Chemotherapy; ⑫: Primary resection; ⑬: Chemotherapy target lesion; ⑭: Capecitabine dosage; ⑮: Rejimen; ⑯: Diarrhea; ⑰: Stomatitis; ⑱: Nausea; ⑲: Anorexia; ⑳: Fatigue; ㉑: Hand-foot syndrome; ㉒:Constipation; ㉓:Leukopenia; ㉔: Neutropenia; ㉕:Anemia; ㉖:Thrombocytopenia

Our analyses revealed a significant difference in the concentration gradient depending on the incidence of diarrhea and nausea. As shown in Figure 2, the heatmap of correlation coefficients among the clinicopathologic factors also showed that the plasma 5-FU concentration gradient correlated with diarrhea and nausea. Although the heatmap also showed a correlation between the plasma 5-FU concentration and hand-foot syndrome, there was no significant relationship between hand-foot syndrome and the plasma 5-FU concentration as shown in Tables 3, 5. On examining the relationship between BSA and AEs, BSA was found to have a significant correlation with fatigue and thrombocytopenia. The relationship between Ccr and AEs was evaluated, and Ccr was found to be correlated with diarrhea (Figure 2).

## Discussion

Several studies have validated the efficacy of 5-FU TDM [4-6,19]. 5-FU dose adjustment by pharmacokinetic monitoring compared to BSA adjustment is associated with better response rates and less severe toxicity. The area under the receiver operating characteristic curve for plasma 5-FU concentration is the most appropriate pharmacokinetic parameter associated with 5-FU-associated toxicity and efficacy. For plasma 5-FU concentrations, the area under the receiver operating characteristic curve values ranging from 20-24 mg·h/L have been proposed in various studies [4,6]. To address the issue of frequent blood draws for TDM, the number of blood draws was reduced to two to determine if changes in 5-FU concentration correlated with adverse events. Because plasma 5-FU concentrations peak 1-2 hours after oral capecitabine administration, blood was drawn twice, once at one hour and again at two hours after oral administration, but further study is needed to determine the optimal frequency and time of blood collection. Yoshida et al. reported that plasma 5-FU concentrations after the administration of the 5-FU prodrug capecitabine varied with time, temperature, and the presence of 5-NU, nearly doubling in some combinations of assay conditions [18]. Given that 5-FU has a narrow therapeutic window, accurate measurement of plasma 5-FU concentrations is critical.

In this study, plasma 5-FU concentrations were measured after the addition of 5-NU to the samples. The median plasma concentration, calculated using the higher of the measured 1- and 2-hour 5-FU concentrations, was 0.12 ± 0.06 µg/mL. The median Cmax in a previously conducted study was 0.22 ± 0.12 µg/mL, which was higher than observed in our study [20]. This difference could be attributed to the time of measurement, temperature, and not adding 5-NU to the samples before the measurement.

Diarrhea significantly reduces the quality of life of patients and leads to discontinuation of chemotherapy and refusal to continue treatment [21]. In addition, patients with severe diarrhea are associated with the risk of severe dehydration that can lead to fatal conditions, such as renal failure, electrolyte imbalance, and circulatory failure [21,22]. Furthermore, patients who develop diarrhea during myelosuppression are at risk of secondary sepsis, thereby necessitating appropriate preventive measures [23]. In addition, nausea and vomiting are nonhematologic toxicities that are frequently observed in patients treated with chemotherapeutic drugs [24]. Severe nausea and vomiting often cause anorexia, dehydration, malnutrition, and electrolyte imbalance, leading to decreased compliance or refusal of chemotherapy, which may hinder treatment continuation [25]. Therefore, prior anticipation of these issues and implementation of appropriate preventive measures are important for the continuation of chemotherapy.

After being metabolized to FdUMP by TP and to FUMP by orotate phosphoribosyl transferase, 5-FU exerts antitumor activity by inhibiting deoxyribonucleic acid and ribonucleic acid synthesis, respectively [26]. These metabolites can cause diarrhea in the intestinal tract [27,28]. The high plasma 5-FU concentration gradient observed in the present study suggests that 5-FU may have been more predominantly metabolized to FdUMP and FUMP, leading to intestinal mucosal damage. While plasma concentration of 5-FU is important for the development of adverse events of chemotherapy, the metabolism of 5-FU is also believed to be essential. Although both are useful indicators of adverse events, the plasma concentration of 5-FU was included in this study because the metabolic enzyme activity is consequently reflected in the plasma concentration of 5-FU.

Nausea and vomiting are considered to be caused by the stimulation of the vomiting center, which is located dorsally in the lateral reticular formation of the medulla oblongata [29,30]. There are three known mechanisms of nausea and vomiting associated with chemotherapeutic drugs. The first mechanism involves the direct chemical stimulation of the chemoreceptor trigger zone, located in the area postrema of the fourth ventricle, by the chemotherapeutic drug; the stimulation is then transmitted to the vomiting center. The second mechanism involves the stimulation of the ascending chemoreceptor trigger zone by serotonin secreted by enterochromaffin cells in the gastrointestinal tract. The final mechanism involves the transmission of emotional and sensory stimuli from the cerebral cortex to the vomiting center. The metabolism of 5-FU to FdUMP and FUMP leads to gastrointestinal mucosal damage and release of serotonin from enterochromaffin cells; this mechanism might underlie our finding that the rate of nausea was higher in patients with a high plasma 5-FU concentration gradient.

There were several limitations inherent to the present study.

1) The sample size was relatively small, and this was a single-center study. Thus, multicenter studies are warranted to increase the generalizability of our findings and confirm them in larger cohorts. 2) All patients were prophylactically treated for nausea with antiemetic medications, which might have introduced an antiemetic bias. Third, plasma 5-FU concentrations were measured only twice, and more time points should help in finding the optimal 5-FU concentration gradient with the fewest adverse events. 3) The number of patients with high-grade adverse events was too small to evaluate. 4) The present study is an exploratory study, and a multiplicity of issues may lead to erroneous study results.

Further research would be needed to confirm the present study.

## Conclusions

The plasma 5-FU concentration gradient, defined as the difference between the one- and two-hour concentrations, was higher in patients with diarrhea and nausea. The high plasma 5-FU concentration gradient suggests rapid degradation of 5-FU into its metabolites, which may lead to predicted intestinal mucosal damage, diarrhea, and nausea. Several studies have validated the efficacy of 5-FU TDM, but patients are held longer, and blood samples are drawn more frequently. This study seemed like a good opportunity to develop a method that would reduce the burden on patients.

## Figures and Tables

**Figure 3:**
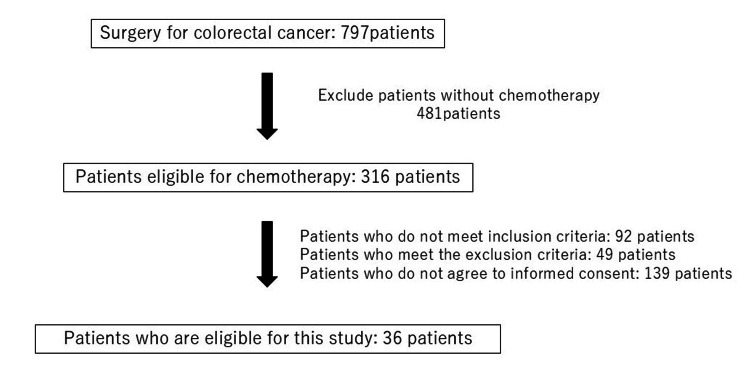
Patient recruitment process The patient recruitment process is shown as supplemental Figure 3.

## Appendices

**Table 6:** The study’s inclusion and exclusion criteria The study’s inclusion and exclusion criteria are shown as supplemental Table 6.

Inclusion criteria	Age: ≥20 years
	Histologically diagnosed colorectal cancer.
	Life expectancy: ≥3 months
	Eastern Cooperative Oncology Group performance status: 0-1
	Neutrophil count: ≥1000/mm^3^
	Hemoglobin: ≥8.0 g /dL
	Platelet count: ≥75,000/mm3
	Serum creatinine: ≤1.5 times the upper limit of normal
	Total bilirubin: ≤2.0 mg /dL
	Aspartate transaminase: ≤100 IU/L (≤200IU/L for patients with metastatic liver tumor)
	Alanine transaminase: ≤100 International Unit (IU) / litre (L) (≤200IU/L for patients with metastatic liver tumor)
Exclusion criteria	Serious drug allergy
	Severe peripheral neuropathy
	Active infection
	Uncontrollable hypertension
	Mechanical or paralytic bowel obstruction
	Uncontrolled diabetes mellitus
	Cirrhosis
	Unstable ischemic heart disease
	Multiple malignancy within the last five years
	Ascites
	Pleural effusion
	Pericardial effusion
	Uncontrolled diarrhea

The patient recruitment process is shown as supplemental Figure 3.
